# Outcomes of Different Quality of Life Assessment Modalities After Breast Cancer Therapy: A Network Meta-analysis

**DOI:** 10.1001/jamanetworkopen.2023.16878

**Published:** 2023-06-06

**Authors:** Stavroula Lila Kastora, Alexander Holmquist, Antonios Valachis, Nicola Rocco, Icro Meattini, Navita Somaiah, Anne Peled, Abhishek Chatterjee, Giuseppe Catanuto, Marios Konstantinos Tasoulis, Maurizio Bruno Nava, Philip Poortmans, Andrea Pusic, Yazan Masannat, Andreas Karakatsanis

**Affiliations:** 1 University College London, UCL EGA Institute for Women’s Health, London, United Kingdom; 2 School of Medicine, Medical Sciences and Nutrition, University of Aberdeen, Aberdeen, United Kingdom; 3 Department of Surgical Sciences, Faculty of Medicine, Uppsala University, Uppsala, Sweden; 4 Department of Oncology, Faculty of Medicine and Health, Örebro University, Örebro, Sweden; 5 Department of Advanced Biomedical Sciences, University of Naples Federico II, Naples, Italy; 6 G.RE.T.A. Group for Reconstructive and Therapeutic Advancements, Milan, Naples, and Catania, Italy; 7 Department of Experimental and Clinical Biomedical Sciences M. Serio, University of Florence, Florence, Italy; 8 Radiation Oncology Unit, Oncology Department, Florence University Hospital, Florence, Italy; 9 Division of Radiotherapy and Imaging, Institute of Cancer Research, London, United Kingdom; 10 Sutter Health California Pacific Medical Center, San Francisco; 11 Division of Surgical Oncology and Plastic Surgery, Tufts Medical Center, Boston, Massachusetts; 12 Humanitas, Institute Clinico Catanese–Misterbianco, Catania, Italy; 13 Breast Unit, Royal Marsden NHS Foundation Trust, London, United Kingdom; 14 Department of Radiation Oncology, Iridium Netwerk, Wilrijk-Antwerpen, Belgium; 15 Faculty of Medicine and Health Sciences, University of Antwerp, Wilrijk-Antwerpen, Belgium; 16 Division of Plastic and Reconstructive Surgery, Brigham and Women’s Hospital, Boston, Massachusetts; 17 Aberdeen Royal Infirmary, Breast Surgery, NHS Grampian, Aberdeen, United Kingdom; 18 Section for Breast Surgery, Department of Surgery, Uppsala University Hospital (Akademiska), Uppsala, Sweden

## Abstract

**Importance:**

Improvement in clinical understanding of the priorities of patients with breast cancer (BC) regarding postoperative aesthetic outcomes (AOs) is needed.

**Objective:**

To assess expert panel and computerized evaluation modalities against patient-reported outcome measures (PROMs), the gold standard of AO assessment, in patients after surgical management of BC.

**Data Sources:**

Embase, MEDLINE, PsycINFO, PubMed, the Cochrane Central Register of Controlled Trials, the World Health Organization International Clinical Trials Registry Platform, and ClinicalTrials.gov were interrogated from inception through August 5, 2022. Search terms included *breast conserving* AND *aesthetic outcome* AND *breast cancer*. Ten observational studies were eligible for inclusion, with the earliest date of database collection on December 15, 2022.

**Study Selection:**

Studies with at least 1 pairwise comparison (PROM vs expert panel or PROM vs computerized evaluation with Breast Cancer Conservation Treatment cosmetic results [BCCT.core] software) were considered eligible if they included patients who received BC treatment with curative intent. Studies reporting solely on risk reduction or benign surgical procedures were excluded to ensure transitivity.

**Data Extraction and Synthesis:**

Two independent reviewers extracted study data with an independent cross-check from a third reviewer. The quality of included observational studies was assessed using the Newcastle-Ottawa Scale, and the level of evidence quality was assessed using the Grading of Recommendations Assessment, Development and Evaluation tool. Confidence in network meta-analysis results was analyzed with the Confidence in Network Meta-analysis semiautomated tool. Effect size was reported using random-effects odds ratios (ORs) and cumulative ratios of ORs with 95% credibility intervals (CrIs).

**Main Outcomes and Measures:**

The primary outcome of this network meta-analysis was modality (expert panel or computer software) discordance from PROMs. Four-point Likert responses across PROMs, expert panel assessment, and BCCT.core evaluation of AOs were assessed.

**Results:**

A total of 10 observational studies including 3083 patients (median [IQR] age, 59 [50-60] years; median [range] follow-up, 39.0 [22.5-80.5] months) with reported AOs were assessed and homogenized in 4 distinct Likert response groups (*excellent*, *very good*, *satisfactory*, and *bad*). Overall network incoherence was low (χ^2^_2_ = 0.35; *P* = .83). Overall, panel and software modalities graded AO outcomes worse than PROMs. Specifically, for *excellent* vs all other responses, the panel to PROM ratio of ORs was 0.30 (95% CrI, 0.17-0.53; *I*^2^ = 86%) and the BCCT.core to PROM ratio of ORs was 0.28 (95% CrI, 0.13-0.59; *I*^2^ = 95%), while the BCCT.core to panel ratio of ORs was 0.93 (95% CrI, 0.46-1.88; *I*^2^ = 88%).

**Conclusions and Relevance:**

In this study, patients scored AOs higher than both expert panels and computer software. Standardization and supplementation of expert panel and software AO tools with racially, ethnically, and culturally inclusive PROMs is needed to improve clinical evaluation of the journey of patients with BC and to prioritize components of therapeutic outcomes.

## Key Points

**Question:**

How concordant are currently available tools for aesthetic outcome (AO) assessment after locoregional therapy for breast cancer (BC)?

**Findings:**

In this network meta-analysis of 10 observational studies including 3083 patients with BC who received surgical treatment, expert panel–based and computer-based AO evaluation consistently scored lower than patient-perceived outcomes.

**Meaning:**

These findings suggest that standardization, adaptation, and dissemination of available AO tools are needed to improve clinical perceptions of the journey of patients with BC, meet patients’ needs, and refine therapeutic outcome priorities.

## Teaser

This network meta-analysis compares expert panel and computerized evaluation modalities with patient-reported outcome measures of aesthetic outcomes after surgical treatment among patients with breast cancer.

## Introduction

With the increasing incidence of breast cancer (BC), a variety of therapeutic advances have been developed to accommodate patient- and disease-tailored safe locoregional treatment management.^1^ In terms of BC surgical management, patient-reported outcomes (PROs) are the primary means of aesthetic and functional outcome quantification of locoregional BC treatment. Multiple approaches have been explored to standardize assessment of the inherently subjective aesthetic outcome (AO) after BC therapy. Various validated tools, such as the BREAST-Q assessment,^2^ the Breast Cancer Treatment Outcome Scale,^3^ and the Hoeller scale,^4^ have been developed to provide a structured framework of PRO measures (PROMs). To that end, the correlation of perceived AO has been explored in the context of health care professional perceptions compared with patient views.^5^ In turn, panel AO assessment tools (Validated Breast Aesthetic Scale^6^ and Fehlauer questionnaire^7^) have been established in the context of standardization. Computerized tools have been designed to minimize subjective AO assessment.^8^

Given the variability of AO assessment modalities, equally variable nonstandardized literature exists regarding the optimal approach to AO evaluation. This variability results in lack of standardization in this field, which, in turn, results in uncertainty regarding whether these different modalities could function as surrogates of each other, complete one another, or serve different roles in the complex issue of tailored treatment and patient-centered care as well as the standardization of clinical practice outcomes. The aim of the present network meta-analysis (NMA) was to assess expert panel and computerized evaluation modalities against PROMs, the gold standard of AO assessment in patients, after surgical management of BC.

## Methods

### Search Strategy and Selection Criteria

**Figure 1.**
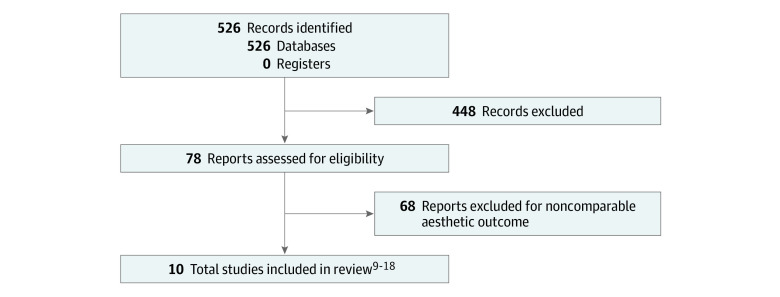
Study Flowchart

This study was registered with PROSPERO (CRD42022382916). Seven databases, comprising Embase, MEDLINE, PsycINFO, PubMed, the Cochrane Central Register of Controlled Trials, the World Health Organization International Clinical Trials Registry Platform, and ClinicalTrials.gov, were searched from inception to August 5, 2022. Search terms included *breast conserving* AND *aesthetic outcome* AND *breast cancer*. Database search and data reporting were performed according to the Meta-analysis of Observational Studies in Epidemiology (MOOSE) reporting guideline. Literature search and study inclusion were conducted according to the Preferred Reporting Items for Systematic Reviews and Meta-analyses (PRISMA) reporting guideline (Figure 1).

Studies with at least 1 pairwise comparison (PROM vs expert panel or PROM vs computerized evaluation with Breast Cancer Conservation Treatment cosmetic results [BCCT.core] software) were considered eligible if they included patients who received BC treatment with curative intention.^9,10,11,12,13,14,15,16,17,18^ Studies reporting solely on risk reduction or benign surgical procedures were excluded to ensure transitivity. Our primary outcome was modality (expert panel and BCCT.core software) discordance from PROMs.

### Additional Measures

Patient demographic characteristics (age, body mass index [calculated as weight in kilograms divided by height in meters squared], and menopausal status), tumor characteristics (size, type, stage, and area affected), treatment approach (operation, lymph node sampling vs clearance, and chemotherapy and/or radiotherapy), and interobserver AO variability (medians and IQRs as reported in individual studies) were extracted, when possible, to assess PRO variability across studies and perform meta-regression analyses for pairwise comparisons. Aggregate data of additional measures were analyzed to provide summative population characteristics.

### Data Extraction and Synthesis

Two independent reviewers (S.L.K. and A.H.) extracted study data with an independent cross-check from a third reviewer (A.K.). After review of the extracted data, data harmonization was performed as follows: (1) all AOs were normalized into a 4-point Likert scale^19^ to allow comparability and homogeneity; (2) Likert scale outcome responses were pooled as *excellent*, *very good*, *satisfactory*, or *bad*; (3) all data from PRO AO reporting tools (BREAST-Q assessment^2^; Hoeller scale^4^; European Organisation for Research and Treatment of Cancer Quality of Life Questionnaire, core and BC-specific versions^20^; EuroQol 5-dimension 5-level questionnaire^21^; and Breast Cancer Treatment Outcome Scale^3^) were pooled together per Likert scale; and (4) results of panel AO reporting tools (Fehlauer questionnaire,^7^ Harris scale,^22^ Validated Breast Aesthetic Scale,^23^ and visual analog scale) were similarly homogenized and pooled for comparability (eTable 1 in Supplement 1). Because a single computerized AO reporting system (BCCT.core) was used across eligible studies, homogenization was not required for this category.^24^ The ordinal data were then dichotomized as recommended by *Cochrane Handbook for Systematic Reviews of Interventions*^25^ (hereafter, Cochrane) methods (eTables 2 and 3 in Supplement 1).

Two intergroup comparisons were formulated: (1) Likert response of *excellent* vs all other responses and (2) Likert responses of *excellent* and *very good* vs *satisfactory* and *bad* (eTable 1 in Supplement 1). We performed a bayesian NMA of results from PROM-controlled and expert panel observational studies vs BCCT.core observational studies (eTables 4-6 in Supplement 1). The quality of included observational studies was assessed using the Newcastle-Ottawa Scale^26^ (eTable 7 in Supplement 1). The certainty of evidence in the quality level was assessed using the Grading of Recommendations Assessment, Development and Evaluation (GRADE) tool^27^ (eTable 7 in Supplement 1). Confidence in NMA results was analyzed with the Confidence in Network Meta-analysis semiautomated tool^28^ (eTable 8 in Supplement 1).

### Qualitative Data Synthesis

After completion of the NMA results, an international panel of breast surgeons, plastic surgeons, and radiation oncologists was invited to discuss the findings, define knowledge gaps, and identify future research priorities. The panelists were selected on the basis of relevance and expertise in the field. They were presented with the results of the NMA together with the confidence in the existing evidence and responded using a preformatted item list^29^ (eAppendix in Supplement 1) in a first round, without knowing the identity of the other members of the panel. Because most questions (questions 2, 3, and 5-16) were of ordinal nature, cumulative replies were visualized as bar charts using GraphPad Prism software, version 9 (GraphPad Software Inc). Qualitative data (questions 4, 17, and 18) were coded and normalized to produce homogeneous discussion points. A single questionnaire was sent to the expert panel (eAppendix in Supplement 1). In questions 2 through 16, we asked the experts to provide their opinion, prior to seeing the findings of the meta-analysis (first round). With regard to question 17, we ask for the personal thoughts of each panel member on the findings of the meta-analysis (second round). Finally, in question 18, we asked for the panelists’ opinion, based on their expertise and the present findings.

### Statistical Analysis

The selected effect size was defined by the random-effect odds ratio (OR) and the cumulative ratios of ORs with 95% credibility intervals (CrIs). The NMA iterations were conducted using 2 Cochrane-developed software programs (netmeta [NMAstudio]^30^ and the MetaInsight visual package for R software, version 4.1 [R Foundation for Statistical Computing]^31^) to ensure cross-platform data validity. Heterogeneity was explored and reported as *I*^2^ values, while network incoherence was reported as χ^2^ values (with *df* and *P* values). The threshold for statistical significance was 2-tailed *P* < .05. Gelman network convergence,^32^ network deviance, and ranking analyses were conducted to quantify overall network discordance (eFigures 1 and 2 in Supplement 1).

## Results

### Design of Included Studies and Quality of Evidence Assessment

A total of 10 observational studies^9,10,11,12,13,14,15,16,17,18^ were deemed eligible for inclusion (Figure 1), with the earliest date of database collection on December 15, 2022. Of note, we could not identify whether randomization was performed in the prospective studies based on the available literature. Individual studies were assessed for quality according to Newcastle-Ottawa Scale items; 8 studies^11,12,13,14,15,16,17,18^ were of good quality, while 2 studies^9,10^ were of fair quality (eTable 7 in Supplement 1). Based on GRADE assessment, confidence in evidence was high for most studies,^11,12,13,14,15,16,17^ while low confidence in evidence was attributed to 1 study^18^ (eTable 8 in Supplement 1). Of those, 5 studies^11,13,14,17,18^ reported κ as effect size, 2 studies^9,12^ were built on regression models, 2 studies^10,15^ reported intraclass correlation coefficients, and 1 study^16^ reported Cronbach α. In 3 studies,^10,15,17^ measures of dispersion (SEs or 95% CIs) were not provided (eTable 2 in Supplement 1). Moreover, different PROs and panel items were assessed across different studies.

### Data Synthesis and Network Meta-analysis

**Figure 2.**
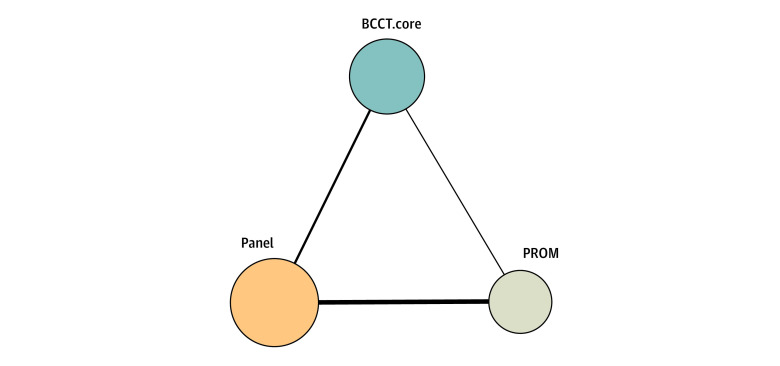
Aesthetic Outcome Modality Network The thickness of edges represents the number of studies involved in the direct modality comparison. The size of aesthetic outcome (AO) modality nodes represents the number of participants assessed per AO tool. BCCT.core indicates Breast Cancer Conservation Treatment cosmetic results software; and PROM, patient-reported outcome measure.

A total of 3083 patient AOs were assessed in the source studies^9,10,11,12,13,14,15,16,17,18^ (eTable 2 in Supplement 1). The median (IQR) age was 59 (50-60) years, and the median (range) follow-up period was 39.0 (22.5-80.5) months. Smoking status was not reported in any of the included studies. Only 2 studies^9,16^ reported postmenopausal status across included patient populations (median [range], 56.2% [45.0%-67.3%]). Medical and surgical management of included patients and tumor grading and histopathological characteristics were not consistently reported across included studies (eTables 3 and 4 in Supplement 1). Regarding NMA AO modality assessment categorizations, 2111 patients reported outcomes,^9,10,12,13,15,16^ 1399 patient images were assessed by an expert panel,^9,10,11,13,14,15,16,18^ and 961 anterior-posterior patient images were assessed by BCCT.core^10,11,12,14,15,17,18^ (eTable 2 in Supplement 1). A diagram of the AO modality network is provided in Figure 2.

**Figure 3.**
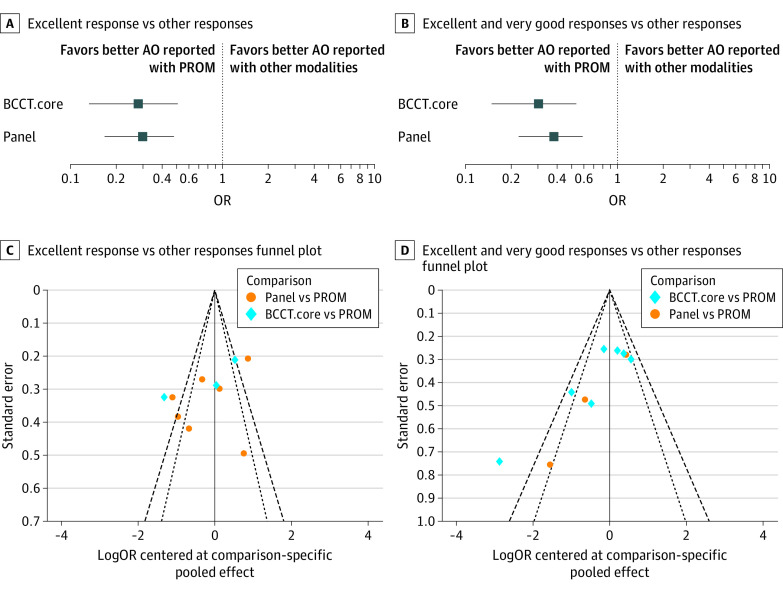
Likert Response Groupings A and B, horizontal lines represent 95% CIs. AO indicates aesthetic outcome; BCCT.core, Breast Cancer Conservation Treatment cosmetic results software; OR, odds ratio; and PROM, patient-reported outcome measure.

In the NMA, effect size estimates suggested that both modalities (expert panel and computer software) consistently undergraded AOs in comparison with PROMs, regardless of Likert scale groupings (Figure 3; eTable 1 in Supplement 1). In turn, the computerized AO tool underrated AOs in comparison with expert panels, although the difference was not significant in any of the Likert groupings (Figure 3). Overall, the NMA confidence rating was high (eTable 8 in Supplement 1).

More specifically, for Likert groupings of *excellent* responses vs all other responses, overall network incoherence was found to be low (*χ^2^*_2_ = 0.35; *P* = .83) with a panel to PROM ratio of ORs of 0.30 (95% CrI, 0.17-0.53) and high subgroup heterogeneity (*I*^2^ = 86%). The BCCT.core to PROM ratio of ORs was 0.28 (95% CrI, 0.13-0.59) with high subgroup heterogeneity (*I*^2^ = 95%), while the BCCT.core to panel ratio of ORs was 0.93 (95% CrI, 0.46-1.88) with high subgroup heterogeneity (*I*^2^ = 88%) (Figure 3; eFigure 1 and eTables 5 and 6 in Supplement 1). For Likert groupings of *excellent* and *very good* responses vs all other responses, overall network incoherence was also low (χ^2^_2_ = 0.34; *P* = .84) (Figure 3). Specifically, the panel to PROM ratio of ORs was found to be 0.32 (95% CrI, 0.18-0.59) with moderate subgroup heterogeneity (*I*^2^ = 71%), the BCCT.core to PROM ratio of ORs was 0.61 (95% CrI, 0.13-2.78) with low subgroup heterogeneity (*I*^2^ = 48%), and the BCCT.core to panel ratio of ORs was 0.91 (95% CrI, 0.50-1.65) with moderate subgroup heterogeneity (*I*^2^ = 73%) (Figure 3; eFigure 1 and eTables 5 and 6 in Supplement 1). Interobserver variability could not be assessed because effect sizes were either underreported or inconsistent across studies (eTable 2 in Supplement 1).

### Expert Panel Questionnaire

**Figure 4.**
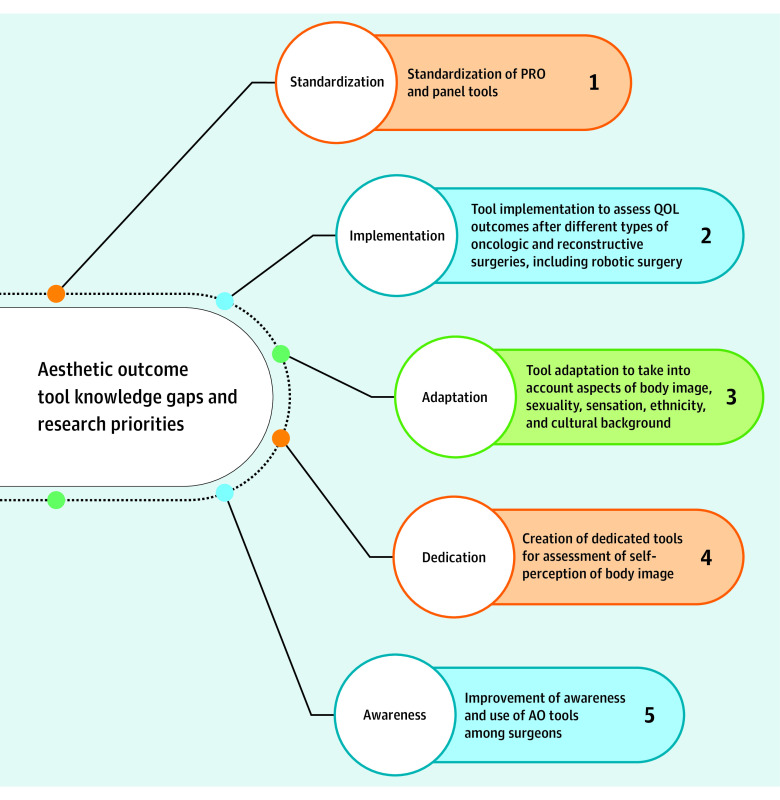
Expert Panel Research Priorities for Existing Aesthetic Outcome Tool Improvement and Knowledge Gaps AO indicates aesthetic outcome; PRO, patient-reported outcome; and QOL, quality of life.

Ten experts were selected based on expertise and engagement in the topic of AO assessment after locoregional BC therapy. These experts were asked to provide their personal opinions regarding current AO tool applicability (questions 2-16) (eFigures 2-4 and eAppendix in Supplement 1), their interpretation of NMA data (question 17), and their views regarding current knowledge gaps and research priorities (question 18) (Figure 4).

Most of the panelists (6 [60%]) felt that existing AO scoring tools are complementary and therefore individually irreplaceable (eFigure 2A and B and eAppendix in Supplement 1). While subjective and possibly dependent on variables such as postoperative pain, chest wall and upper limb morbidity, and toxic effects of systemic treatment, PROMs were considered the most valuable measure of AOs (eFigure 2C in Supplement 1). In addition, while individually irreplaceable, a degree of overlap was felt to exist between the 3 modalities, the least profound of which was felt to be between PROM responses and software AO assessment (eFigure 2D in Supplement 1). The expert panel appeared to favor PROMs as the gold standard of AO assessment for guiding preoperative decision-making in view of surgical technique (6 panelists [60%]), evaluating outcomes of primary surgical treatment (7 panelists [70%]), and guiding decisions in view of revision procedures (8 panelists [80%]), while 5 panelists (50%) favored PROMs as means of assessing postradiotherapy outcomes (eFigure 3A-D in Supplement 1). Most panelists (8 [80%]) considered PROMs to be the modality of choice for evaluating outcomes after revision procedures and reporting AOs in clinical research, and they believed PROMs were the optimal tools for enabling trainees to comprehend and systematically evaluate AO outcomes after BC surgical procedures (eFigure 3A-C in Supplement 1). Overall, 8 panelists (80%) favored PROMs as the tools of choice in reporting AOs in clinical practice, setting PROMs as the single most preferred modality to comprehend AOs and guide clinical practice (eFigure 4A-D in Supplement 1). Regarding free-text replies to question 18 (eAppendix in Supplement 1), 5 panelists (50%) felt that there is potential for improvement through standardization of, optimally, a single validated PRO questionnaire, while 3 panelists (30%) felt that a similar standardization would be necessary to improve panel AO reporting, and 2 panelists (20%) felt that software-based standardization in terms of PROM and panel assessment integration was needed (Figure 4).

## Discussion

Considering the observational design and the marked discordance of the meta-synthesized studies,^9,10,11,12,13,14,15,16,17,18^ a uniform finding of the present NMA was that both expert panel–based and computer-based evaluations consistently scored AOs lower than patient-perceived outcomes. In agreement with available literature,^33,34,35,36^ PROMs can be considered the reference standard of AO assessment but not a surrogate for other AO scoring tools, either panel or computer based.

More specifically, regarding the NMA outcomes, the direct comparisons were obtained from 3 observational studies^11,15,17^ with potential bias. In general, PROMs are the current gold standard. They are, by definition, patient centered and allow for insight on patient experience. Equally, the challenges of PROM interpretation are well known, as the outcomes are more likely to reflect a personal perspective that is, by nature, complex and multifactorial, despite the fact that the items are designed and validated to be specific.^33,34^ Conversely, tools for panel- or computer-based reporting are based on the belief that an external reviewer or a computerized assessment of photographic measurements is expected to be objective. However, none of these modalities has been supplemented with experiential patient data, which, despite their subjective nature, have substantial implications for the overall outcome perception, in turn generating the noted level of discordance across modality outcomes.^35,36,37^

As also reflected across responses to the expert panel questionnaire, PROMs are more complex in their interpretation and may be substantially affected by the entirety of the patient’s journey rather than solely by the surgical outcome.^36,37^ In that sense, some of the factors that may play a role in PRO AO reporting, such as disease- and treatment-related adverse effects (eg, malaise, hair loss, and loss of sensation), baseline treatment expectations, financial toxicity, patient race and ethnicity, patient cultural background, and body image perception and sexuality, are underrepresented in all AO modalities and therefore quantifiably elusive.^33,34,37^ On the other hand, expert panels may be composed of different stakeholders with different levels of knowledge, as reflected across the included studies^9,10,11,12,13,14,15,16,17,18^; therefore, preconceptions of AOs may increase the variability of responses.^33^ In addition, while software-based assessment of AO images may control to a degree the inherent level of perception bias, one should be mindful that data taken into consideration reflect a visual result that may not detect aspects such as texture, sense, and symptoms.^33^ Overall, despite the subjectivity of PROMs and their largely experiential nature, they represent the benchmark of AO assessment, as unanimously supported by the expert panel, and incorporate the essence of patient-centered and patient-led care. In that sense, a novel combinatorial approach toward AO assessment that enhances the strengths and ameliorates the weaknesses of each individual tool may be needed.

In addition to AO scoring tool standardization, augmentation of available tools to incorporate aspects of patient race and ethnicity, cultural influences, sexuality, and self-views of body image would substantially improve the perspective necessary to holistically assess surgical outcomes.^35,37,38^ Efforts to develop such tailored tools have been undertaken, especially in the context of race and ethnicity, but expert consensus appraisal and standardization of these tools remains to be achieved.^39^ Furthermore, adjustment of AO tools to the specific surgical approach undertaken (eg, robotic vs open nipple-sparing mastectomy) would set a realistic benchmark for AO expectations and therefore outcome assessment.^35,36,38^ As an adjunct to the development of optimized AO tools in future research, dedicated and standardized body image tools in BC should be developed to provide pragmatic and all-inclusive views on preestablished body perceptions in this patient group (Figure 4).

Our findings suggest that the development of validated standardized AO tools tailored to patients with BC is needed to enhance the quality and accuracy of AO reporting in both the clinical and research settings, comprehensively guide clinical management, and ultimately improve the patient experience. Because PRO reporting tools such as the BREAST-Q have not been validated in different populations, whether racial, ethnic, and cultural factors play a role in PROs is not known, which in turn constitutes a meaningful gap in current AO assessment and necessitates future studies to evaluate differences between PROMs depending on patient race and ethnicity, sexuality, and specific surgical approach.^40,41,42^ Even with substantial financial and research investments, modifications may not be successful if they are not widely adopted and used in the clinical setting. Therefore, improving awareness of AO tools across clinicians is equally, if not more, important to reimagining all-inclusive AO tools that are able to incorporate the diversity of the modern patient’s body image^43,44^ (Figure 4). To that end, with the increase in e-learning environments and social media platforms in recent years, it seems reasonable to consider that the use of AO tools may be disseminated rapidly through the static web (ie, web 1.0, comprising read-only static web pages providing information) and the social media web (ie, web 2.0, comprising dynamic and participatory user-generated content) in the context of surgical training and education.^43,44,45^ Online information hubs for patients and health care professionals already exist across social media; these hubs may be used as conduits for intergroup knowledge flow and to stimulate a productive dialogue with the aim of achieving a balance between safe cancer treatment and optimal AOs for patients.^40,41,42^

### Limitations

This study has several limitations. Despite all efforts to minimize data bias, inherent limitations of the present work stem from the retrospective and observational design of available studies^9,10,11,12,13,14,15,16,17,18^ as well as the underreporting of important variables, such as patient demographic characteristics, disease characteristics, and AO interobserver variability. In addition, there is an inherent limitation with potential bias present when normalizing and converting AO data from the expert panel^9,10,11,13,14,15,16,18^ and PROM^9,10,12,13,15,16^ studies into a 4-point Likert scale to homogenize the data and facilitate comparisons between the groups being studied. To that end, single-study and meta-synthesized evidence were assessed by different groups of reviewers to ensure objectivity and transparency of evidence appraisal. Furthermore, a diverse (with regard to sex, race and ethnicity, and geographic location) panel of experts in their respective fields (breast surgery, plastic and reconstructive surgery, and radiotherapy) was assembled to provide insight and comment on the findings of the meta-analysis in 2 predefined stages, thereby independently controlling for data interpretation bias. As uniformly identified by the invited expert panel, the types of tools implemented across studies were different, which in turn requires that the results should be interpreted in the context of this restriction. To address these inherent study limitations, expert views were quantitatively and qualitatively analyzed to generate author-independent views on present knowledge gaps and future research perspectives.

## Conclusions

In this NMA of AO tool outcomes, patients scored AOs higher than both expert panels and computer software. Given the consistent underestimation of AOs by both expert panels and previously validated software compared with patient appraisal, further studies to define a multimodal approach of AO scoring, benchmarked against the gold standard of patients’ own AO assessments, should be pursued. In that effort, PROMs should remain the core of the novel integrated AO assessment tool to allow the independent voice of patients to become systemically integrated and in turn shape future clinical practice.

## Notes

Supplement 1. **eFigure 1.** Likert Groupings *Excellent* vs All Other Responses (A-D) and *Excellent* and *Very Good* vs All Other Responses (E-H), Bayesian Analysis **eFigure 2****.** Node-Splitting Model of *Excellent* vs All Other Responses (Figure 3A) and *Excellent* and *Very Good* vs All Other Responses (Figure 3B) **eFigure 3.** Expert Panel Responses to Ranking Questions, From Most to Least Important, Questions 7 to 10 **eFigure 4****.** Expert Panel Responses to Ranking Questions, From Most to Least Important, Questions 11 to 14 **eTable 1.** Likert Reporting per Original Publication and Normalized Categories to Facilitate Cumulative Data Analysis **eTable 2.** PICO Chart of Included Studies **eTable 3.** Aggregate Patient Data Regarding Medical and Surgical BC Management **eTable 4.** Aggregate Patient Data Regarding Tumor Characteristics **eTable 5.** AO Modality Network Ratio of ORs and Incoherence Statistical Significance of Comparison per Outcome **eTable 6.** Node-Splitting Model of *Excellent* vs All Other Responses (Figure 3A) and *Excellent* and *Very Good* vs All Other Responses (Figure 3B), Supplementary to Figure 2 **eTable 7.** Newcastle-Ottawa Scale and GRADE Rating per Study **eTable 8.** CiNEMa NMA Ratings **eAppendix.** Expert Panel Questionnaire
eReferences

Supplement 2.
Data Sharing Statement
