# Supplementary material for: Outcomes of Different Quality of Life Assessment Modalities After Breast Cancer Therapy: A Network Meta-analysis
Source: JAMA Netw Open. 2023 Jun 6;6(6):e2316878. doi: 10.1001/jamanetworkopen.2023.16878 (PMC10245190; doi:10.1001/jamanetworkopen.2023.16878)
Supplement: Supplement 1. — eFigure 1. Likert Groupings Excellent vs All Other Responses (A-D) and Excellent and Very Good vs All Other Responses (E-H), Bayesian Analysis eFigure 2. Node-Splitting Model of Excellent vs All Other Responses (Figure 3A) and Excellent and Very Good vs All Other Responses (Figure 3B) eFigure 3. Expert Panel Responses to Ranking Questions, From Most to Least Important, Questions 7 to 10 eFigure 4. Expert Panel Responses to Ranking Questions, From Most to Least Important, Questions 11 to 14 eTable 1. Likert Reporting per Original Publication and Normalized Categories to Facilitate Cumulative Data Analysis eTable 2. PICO Chart of Included Studies eTable 3. Aggregate Patient Data Regarding Medical and Surgical BC Management eTable 4. Aggregate Patient Data Regarding Tumor Characteristics eTable 5. AO Modality Network Ratio of ORs and Incoherence Statistical Significance of Comparison per Outcome eTable 6. Node-Splitting Model of Excellent vs All Other Responses (Figure 3A) and Excellent and Very Good vs All Other Responses (Figure 3B), Supplementary to Figure 2 eTable 7. Newcastle-Ottawa Scale and GRADE Rating per Study eTable 8. CiNEMa NMA Ratings eAppendix. Expert Panel Questionnaire eReferences [file jamanetwopen-e2316878-s001.pdf]

Supplementary Online Content

Kastora SL, Holmquist A, Valachis A, et al. Outcomes of different quality of life assessment modalities after breast cancer therapy: a network meta-analysis. *JAMA Netw Open*. 2023;6(6):e2316878. doi:10.1001/jamanetworkopen.2023.16878

eFigure 1. Likert Groupings *Excellent* vs All Other Responses (A-D) and *Excellent* and *Very Good* vs All Other Responses (E-H), Bayesian Analysis

eFigure 2. Node-Splitting Model of *Excellent* vs All Other Responses (Figure 3A) and *Excellent* and *Very Good* vs All Other Responses (Figure 3B)

eFigure 3. Expert Panel Responses to Ranking Questions, From Most to Least Important, Questions 7 to 10

eFigure 4. Expert Panel Responses to Ranking Questions, From Most to Least Important, Questions 11 to 14

eTable 1. Likert Reporting per Original Publication and Normalized Categories to Facilitate Cumulative Data Analysis

eTable 2. PICO Chart of Included Studies

eTable 3. Aggregate Patient Data Regarding Medical and Surgical BC Management

eTable 4. Aggregate Patient Data Regarding Tumor Characteristics

eTable 5. AO Modality Network Ratio of ORs and Incoherence Statistical Significance of Comparison per Outcome

eTable 6. Node-Splitting Model of *Excellent* vs All Other Responses (Figure 3A) and *Excellent* and *Very Good* vs All Other Responses (Figure 3B), Supplementary to Figure 2

eTable 7. Newcastle-Ottawa Scale and GRADE Rating per Study

eTable 8. CiNEMa NMA Ratings

eAppendix. Expert Panel Questionnaire

eReferences

This supplemental material has been provided by the authors to give readers additional information about their work.

© 2023 Kastora SL et al. *JAMA Network Open*.

eFigure 1. Likert Groupings *Excellent* vs All Other Responses (A-D) and *Excellent* and *Very Good* vs All Other Responses (E-H), Bayesian Analysis

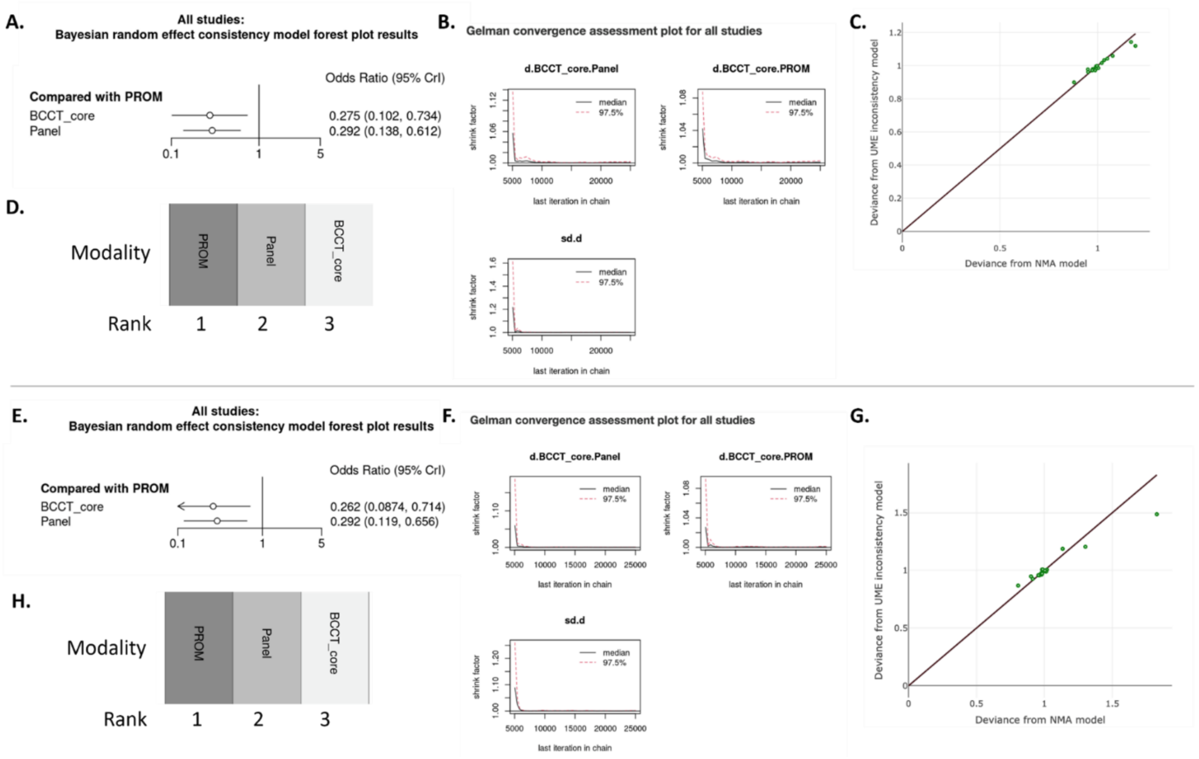

Forest plots where PROM used as reference (A, E), Gelman network convergence (B, F), network deviance plot (C, G) and Ranking analysis (D, H), are also provided. Plots generated with MetaInsight R Package.

© 2023 Kastora SL et al. *JAMA Network Open*.

eFigure 2. Node-Splitting Model of *Excellent* vs All Other Responses (Figure 3A) and *Excellent* and *Very Good* vs All Other Responses (Figure 3B)

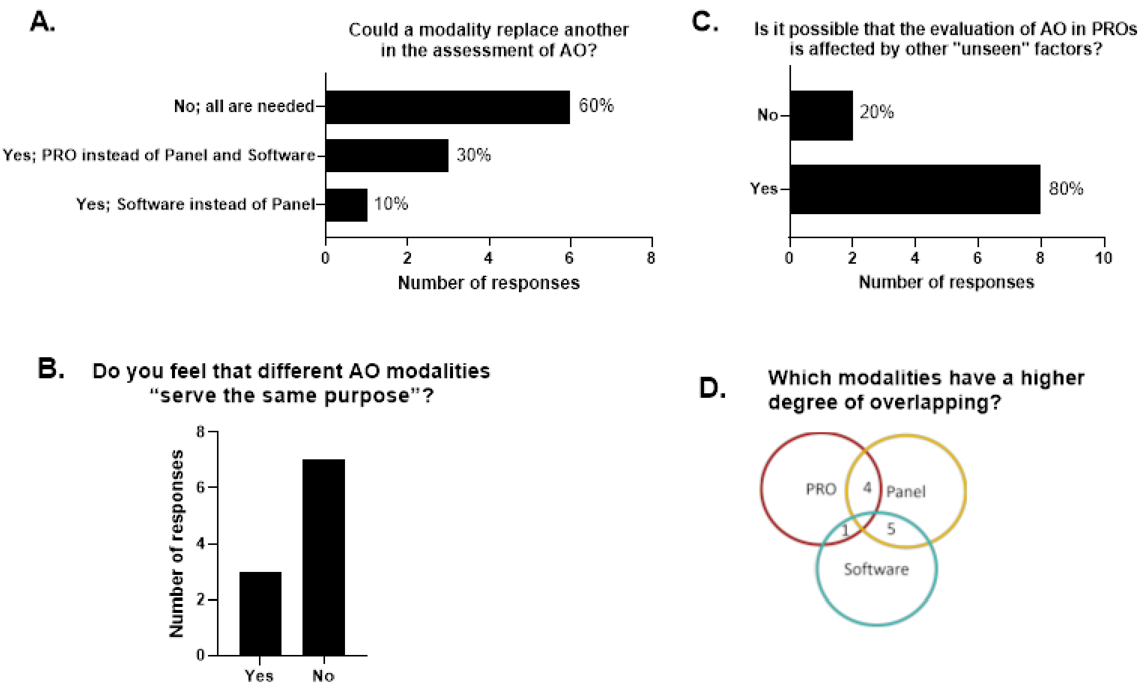

© 2023 Kastora SL et al. *JAMA Network Open*.

eFigure 3. Expert Panel Responses to Ranking Questions, From Most to Least Important, Questions 7 to 10

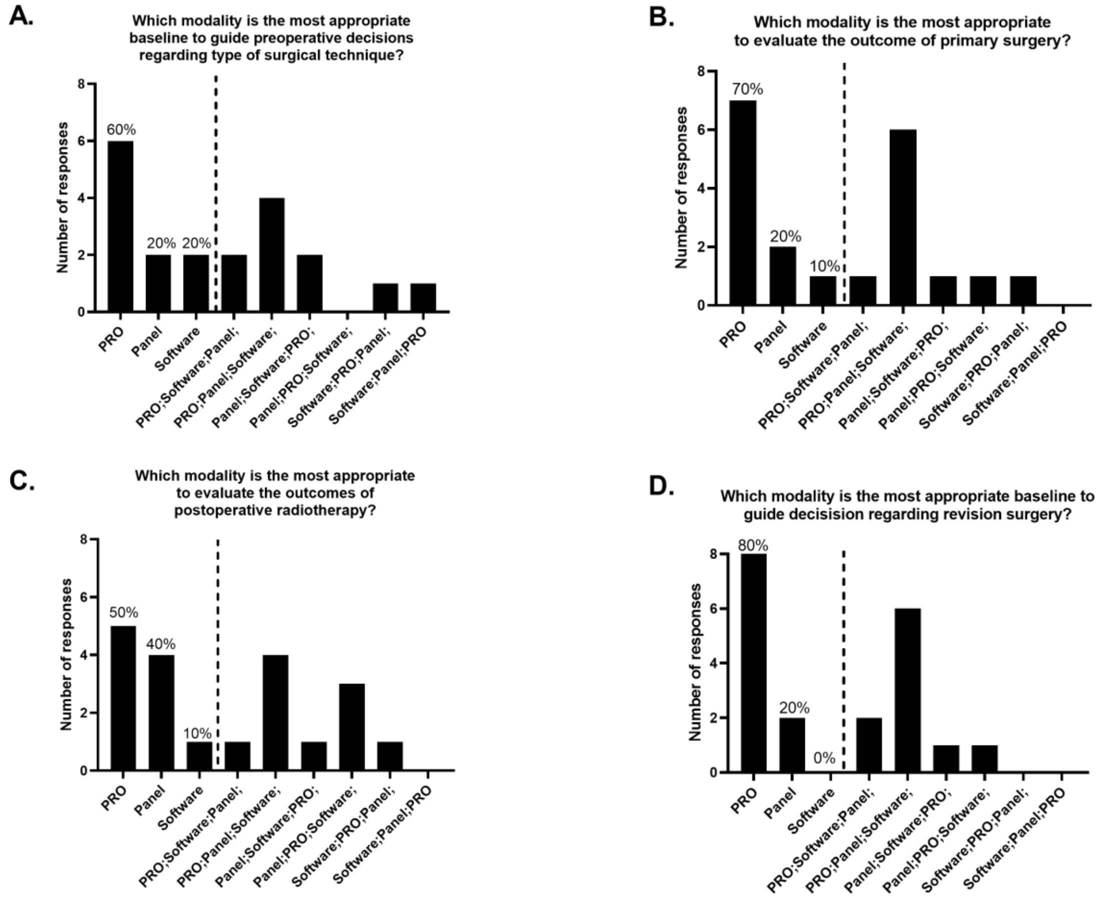

© 2023 Kastora SL et al. *JAMA Network Open*.

eFigure 4. Expert Panel Responses to Ranking Questions, From Most to Least Important, Questions 11 to 14

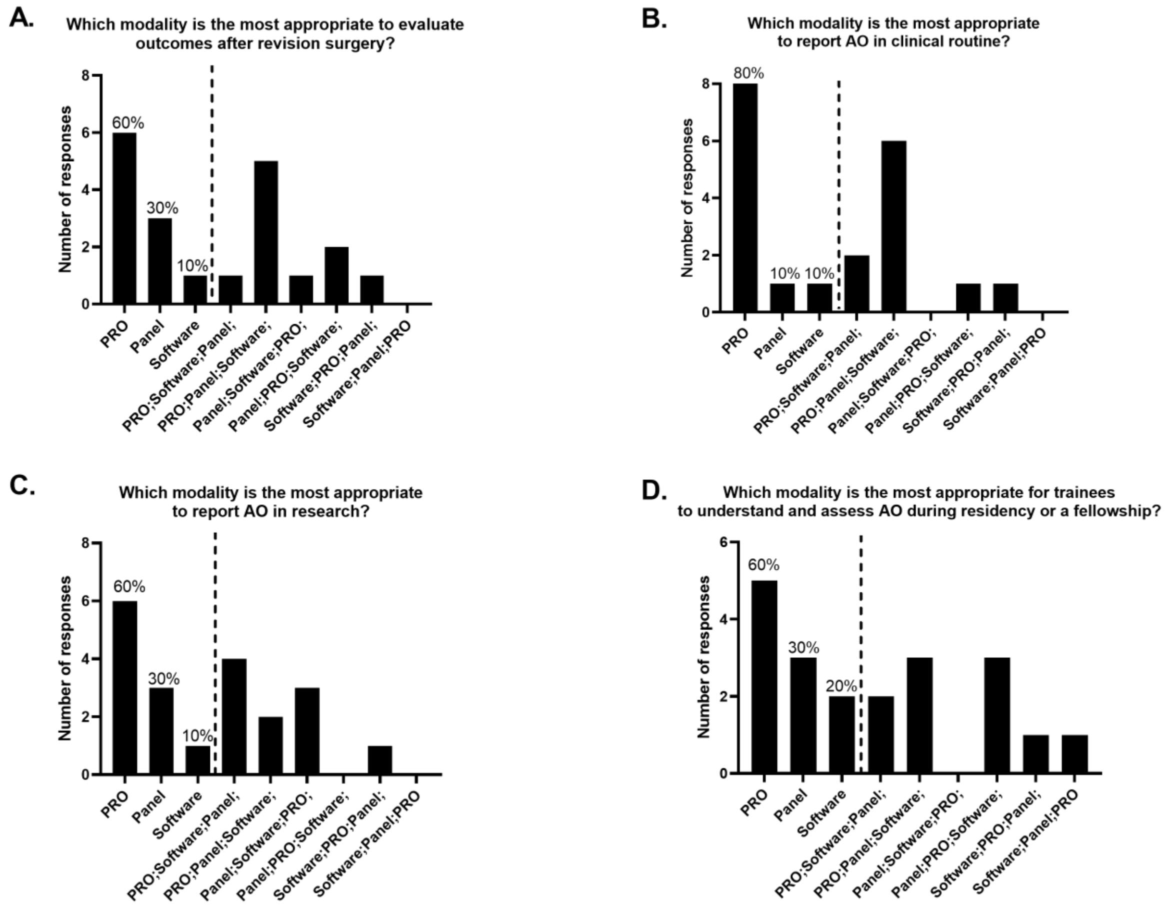

© 2023 Kastora SL et al. *JAMA Network Open*.

eTable 1. Likert Reporting per Original Publication and Normalized Categories to Facilitate Cumulative Data Analysis

A/a	4-Point Likert scale	5-Point Likert scale	5-Point Likert scale normalisation in 4 categories employed in network meta-analysis
Brunault et al., [1] 2013		✓	Likert Response “Excellent” includes “Excellent” category of original publication.<br>Likert response “Very good” includes “Good” category of original publication.<br>Likert response “Satisfactory” includes “Satisfying” category of original publication.<br>Likert response “Bad” includes “Bad” and Very Bad” categories of original publication.
Dahlbäck et al.,[2] 2018	✓		
Haloua et al.,[3] 2014	✓		
Hennigs et al.,[4] 2016	✓		
Kim et al.,[5] 2015	✓		
Waljee et al.,[6] 2008	✓		
Sneeuw et al.,[7] 1992	✓		
Santos et al.,[8] 2015		✓	Likert Response “Excellent” includes “Very Good” category of original publication.<br>Likert response “Very good” includes “Good” category of original publication.<br>Likert response “Satisfactory” includes “Moderate” and “Fair” categories of original publication.<br>Likert response “Bad” includes “Poor” category of original publication.
Wu et al., [9] 2022	✓		
Zwakman et al., [10] 2022		✓	Likert Response “Excellent” includes “5” (PRO) or “>75-100%” (BCCT.core) category of original publication.<br>Likert response “Very good” includes “4” (PRO) or “>50-75%” (BCCT.core) category of original publication.<br>Likert response “Satisfactory” includes “2 and 3” (PRO) or “>25-50%” (BCCT.core) categories of original publication.<br>Likert response “Bad” includes “1” (PRO) or “0-25%” (BCCT.core) categories of original publication.

© 2023 Kastora SL et al. *JAMA Network Open*.

eTable 2. PICO Chart of Included Studies

A/a	Country	Sample size	Operation (Mx_R, BCT)	Follow-up (Median ± SD or range[c]) in months	Comparison	Breast cosmesis assessment method /instrument	Type of effect size	AO [type of effect size, numeric value, SE and in-study p-value]; upk/wk	Panel characteristics
Brunault et al.,[1] 2013	France	120	BCT	80.4 [73.2-132][c]	Hoeller Qs (Patients) vs. Fehlauer (Doctors)	Hoeller et al. Qs[d] _Patients; Fehlauer et al.[d] _Doctors	Cronbach's alpha; Factors influencing outcome Cox regression	Panel vs. PRO 1.7 (95% CI 1.02-2.86; P=0.03) Internal consistency a = 0.58, no P value provided	Number and panel member specialty not stated
Dahlbäck et al.,[2] 2018	Sweden	532	BCT	16 [11 – 23][c]	BCCT.core (N=310) vs Panel (N=215) vs Breast Q (N=348)	Panel[e]; Breast-Q[e]; BCCT.core[e]	Weighted k	Breast Q vs. BCCT.core: 0.65 (Cut off: 66) P= 0.003. Panel vs. BCCT.core_ wk 0·46 [0·43 to 0·60]; P<0.0001	3-member panel (nurse Plastic and Reconstructive Surgery, nurse Breast Surgery, doctor_ general and plastic surgery)

© 2023 Kastora SL et al. *JAMA Network Open*.

Haloua et al.,[3] 2014	Netherlands	109	BCT	20 [12–40] [c]	BCCT.core vs. Panel score	Panel[e]; BCCT.core[e]	Weighted k	Panel vs. BCCT.core OR 1.44 (95% CI 0.84-2.47; P=0.17) Panel vs. BCCT.core_ wk 0.68 (95% CI 0.57-0.77); no P value provided	10- member panel; two breast surgeons, two surgical residents, two laypersons, and four experienced plastic surgeons [f]
Hennigs et al.,[4] 2016	Germany	621	BCT	[24-72][c]	No comparison	BCTOS[e]	Chi square; Factors influencing outcome_log regression (OR, 95% CI)	OR: 2.48; 95% CI: 1.4-12.48); P= 0.008	N/A
Kim et al.,[5] 2015	Korea	617 [a]	BCT N=485 Mx_R N=46,	25.2 [No range provided]	BCCT.core vs. Panel	BCCT.core[e] EORTC QLQ-C30 and the BR23 module	Weighted k; Factors influencing outcome_log regression (OR, 95% CI)	OR (BCCT.core) 5.13; 95% CI 0.04-10.221; P=0.048.<br>OR (Panel) 3.09; 95% CI 1.19-7.38; P=0.015,<br>Panel vs. BCCT.core_ wk 0.35 [95% CI 0.25-0.5; P <0.0001]	4- member panel; physicians[g]

© 2023 Kastora SL et al. *JAMA Network Open*.

Waljee et al.,[6] 2008	USA	635 [b]	BCT	30 [<12-48][c]	No comparison	BCTOS[e]	Factors influencing outcome_log regression (OR, 95% CI)	OR 1.53 (95% CI 0.92-2.56; P= 0.002)	N/A
Sneeuw et al.,[7] 1992	UK	76	BCT	48 [24-132]	PRO vs Panel	Panel[e] vs Patient Qs (Qs as per the present study) [e]	Cohens' k	Patient vs Panel OR 5.54 (95% CI 2.33-13.16; P<0.0001)<br>Panel vs. Patient_ Cohens' k 0.08 (0.07-0.09); no P value	2-member panel; oncology nurse, radiation oncologist
Santos et al.,[8] 2015	Brazil	122	BCT N=65; Mx_R N=57	38.8 [16.4-57.1]	BCCT.core vs. Panel	BCCT.core[e]; Garbay scale[e]	Weighted k	Patient vs Panel OR 1 (95% CI 0.27-3.66; P=1)<br>Patient vs. BCCT. core OR 3.027 (95% CI 1.01-9.29; P=0.04)<br>Panel vs BCCT.core k = 0.12 (Low).<br>Panel vs. Patient_wk not provided.<br>BCCT.core vs. Patient_wk not provided, P<0.001.	4-member panel; 2 breast surgeons and 2 plastic surgeons

© 2023 Kastora SL et al. *JAMA Network Open*.

Wu et al., [9] 2022	USA	147	BCT	Not stated	BCCT.core vs. Panel	BCCT.core[d]; Vibras scale (0-100%)[d]	Pearson R	No OR [95% CI] provided	6-member panel; 2 plastic surgeons, 1 plastics resident, 3 medical school students
Zwakman et al., [10] 2022	Netherlands	104	BCT	78 [72-96]	BCCT.core vs. Panel vs. PROM	BCCT.core[e]; Panel Delgado et al visual scale [e]; Patients (EORTC QLQ-BR23)	Spearman R	Panel vs BCCT.core R: 0.558, P < 0.001 Patients ('body image') vs. BCCT.core R: 0.110, P = 0.267	14-member panel; 3 oncologic surgeons, 3 plastic surgeons, 3 surgery residents and 3 plastic surgery residents and two breast oncology specialized nurses

Mx+ Recon: Mastectomy and Reconstruction; BCT: Breast Conserving Therapy; N/A: Non-applicable, Agreement at follow: upk=kappa; wk: weighted kappa
[a] 87 patients underwent mastectomy, [b]79 patients required further Mastectomy. [c] Marked values indicate range, [d]Measured in 5-point Likert scale, [e] Measured in 4-point Likert scale, [f] at least 10 years of experience with breast reconstruction surgery [g] Specialty not stated

© 2023 Kastora SL et al. *JAMA Network Open*.

eTable 3. Aggregate Patient Data Regarding Medical and Surgical BC Management

Variable	Studies reporting	Percentage of population (%)	Crude Number of patients with reported variable	Total number of patients in studies reporting the given variable	
Chemotherapy	7 out of 10	46.7	1024	2217	
Hormonal Therapy	4 out of 10	59.2	813	1373	
Radiation therapy	7 out of 10	85.8	1917	2235	
Wide local excision or oncoplastic surgery	10 out of 10	91.4	2818	3083	
Mastectomy	10 out of 10	2.8	86	3083	
Axillary node clearance	6 out of 10	6.6	95	1440	

Of the included studies, seven reported whether patients had received chemotherapy, 46.7% [$N_{\text{received chemotherapy}}/N_{\text{reported chemotherapy}}$=1024/2217]. [1-2, 4-5,7, 9-10] Whether patients had received hormonal therapy was reported in four studies 59.2% [$N_{\text{received HT}}/N_{\text{reported HT status}}$ = 813/1373]. [1-2, 5, 10,] Seven studies reported whether patients underwent radiation therapy post-surgical management, 85.8% [$N_{\text{received RT}}/N_{\text{reported RT status}}$ = 1917/2235. [1-2 , 4, 5, 8-10]

© 2023 Kastora SL et al. *JAMA Network Open*.

eTable 4. Aggregate Patient Data Regarding Tumor Characteristics

Tumour characteristics					
Variable	Studies reporting	Mean (cm)	Standard Deviation (cm)		
Tumour size	3 out of 10	1.6	0.15		
Variable	Studies reporting	Tis (%, N/Total)	Stage 1 (%; N/Total)	Stage 2 (%, N/Total)	Stage 3 or 4 (%, N/Total)
Stage	4 out of 10	17.3; 330/1904	35.8; 681/1904	24. 9; 474/1904	2.8; 53/1904
Variable	Studies reporting	Ductal	Lobular	Other	
Histology	4 out of 10	65.4; 357/546	13.2; 72/546	21.4; 117/546	

Tumour size was reported in three studies, mean (cm): 1.6 cm (SD: 0.15). [2-3, 8] Histological categorisation of malignancy was reported for 546 patients, where 65.4% (N=357) were diagnosed with Ductal, 13.2% (N=72) with lobular and 21.4% (N=117) with another histological type. Cancer staging was reported in 4 studies with the majority of patients been diagnosed with Stage 1 cancer, 35.8% ($N_{Stage\ 1}/N_{reported\ Staging}$ =681/1904), 17.3% with carcinoma in situ (Tis) ($N_{Tis}/N_{reported\ Staging}$ =330/1904), 24. 9% T2 ($N_{Stage\ 2}/N_{reported\ Staging}$ =474/1904), 2.73% T3 ($N_{Stage\ 3}/N_{reported\ Staging}$ =52/1904), and 0.05% T4 ($N_{Stage\ 4}/N_{reported\ Staging}$ =1/1904). [1-2, 5-6] Regarding surgical management, approach was reported across all studies with 91.4 % (N=2818) undergoing breast conserving treatment (wide local excision or oncoplastic surgery) and 2.8% (N=86) undergoing mastectomy. Axillary node clearance was undertaken in 95 patients (6.6%) albeit lymph node management was only reported in six studies ($N_{ANC}/N_{reported\ axillary\ operation}$ =95/1440). [2-4, 7, 8, 10]

© 2023 Kastora SL et al. *JAMA Network Open*.

eTable 5. AO Modality Network Ratio of ORs and Incoherence Statistical Significance of Comparison per Outcome

Comparator	"Excellent" vs. all other responses	"Excellent" and "very good" vs. all other responses
Network Incoherence	χ^2 0.35, 2 degrees of freedom, P value: 0.83	χ^2 0.34, 2 degrees of freedom, P value: 0.84
Panel vs. PRO	0.30 [95% CI 0.17 to 0.53]; I^2: 86%	0.32 [95% CI 0.18 to 0.59]; I^2: 71%
BCCT.core vs. PRO	0.28 [95% CI 0.13 to 0.59]; I^2: 95%)	0.61 [95% CI 0.13 to 2.78]; I^2: 48%
BCCT.core vs. Panel	0.93 [95% CI 0.46 to 1.88]; I^2: 88%)	0.91 [95% CI 0.50 to 1.65]; I^2: 73%

© 2023 Kastora SL et al. *JAMA Network Open*.

eTable 6. Node-Splitting Model of *Excellent* vs All Other Responses (Figure 3A) and *Excellent* and *Very Good* vs All Other Responses (Figure 3B), Supplementary to Figure 2

**Design-by-treatment interaction model**								
Outcome	Q	df(Q)	p-value					
Excellent vs. All other	10.693	2	0.0048					
Excellent and Very Good vs.	11.984	2	0.0025					
**Node splitting model**								
Comparison Excellent vs. All other	direct	indirect	p-value		Comparison Excellent and Very Good vs. All other	direct	indirect	p-value
Panel vs PROM	0.3084	0.1069	0.5168		Panel vs PROM	0.3717	0.8626	0.6087
BCCT.core vs PROM	0.2547	0.3696	0.6732		BCCT.core vs PROM	0.2528	0.4204	0.5017
BCCT.core vs Panel	0.9379	0.9319	0.9957		BCCT.core vs Panel	0.8449	0.4436	0.5559

© 2023 Kastora SL et al. *JAMA Network Open*.

eTable 7. Newcastle-Ottawa Scale and GRADE Rating per Study

	Selection				Comparability	Outcome			Max of 9						
	NOS scale										GRADE ratings				
Study	Representativeness of the exposed cohort (Patients)	Selection of the non-exposed cohort (Other means of AO assessment	Ascertainment of exposure	Demonstration that outcome of interest was not present at start of study	Comparability of cohorts based on the design or analysis controlled for confounders	Assessment of outcome	Was follow-up long enough for outcomes to occur	Adequacy of follow-up of cohorts		Notes	Risk of Bias	Imprecision	Inconsistency	Indirectness	Publication bias
Brunault et al., [1] 2013	1	1	1	1	2	1	1	1	9	Nil deducted	Moderate	Moderate	High	High	High
Dahlbäck et al.,[2] 2018	1	1	1	1	2	1	1	1	9	Nil deducted	Moderate	High	Moderate	Moderate	High
Haloua et al.,[3] 2014	0	1	1	1	2	1	1	1	8	1 point off- no patient scoring	Moderate	Very low	Moderate	High	High

© 2023 Kastora SL et al. *JAMA Network Open*.

Hennigs et al.,[4] 2016	0	1	1	1	0	1	1	1	6	2 points off-no comparison, no patient scoring	Moderate	High	Moderate	Low	High
Kim et al.,[5] 2015	0	1	1	1	2	1	1	1	8	1 point off- no patient scoring	Low	High	Moderate	Moderate	High
Waljee et al.,[6] 2008	0	1	1	1	0	1	1	1	6	2 points off-no comparison, no patient scoring	Moderate	Moderate	Moderate	High	High
Sneeuw et al.,[7] 1992	1	1	1	1	2	1	1	1	9	Nil deducted	Moderate	Moderate	High	High	High
Santos et al.,[8] 2015	0	1	1	1	2	1	1	1	8	1 point off- no patient scoring	Moderate	Moderate	High	Moderate	High
Wu et al., [9] 2022	1	1	1	0	2	1	0	1	7	No follow up stated, no raw numbers of	Moderate	High	High	Moderate	High

© 2023 Kastora SL et al. *JAMA Network Open*.

										patient responses					
Zwakman et al.,[10] 2022	1	1	*	*	**	*		*	8		Moderate	High	High	High	High

NOS Rating interpretation: For an overall assessment of good quality 3 or 4 stars in selection domain AND 1 or 2 stars in comparability domain AND 2 or 3 stars in outcome/exposure domain need to be awarded. Equally, Fair quality, 2 stars in selection domain AND 1 or 2 stars in comparability domain AND 2 or 3 stars in outcome/exposure domain and poor quality, 0 or 1 star in selection domain OR 0 stars in comparability domain OR 0 or 1 stars in outcome/exposure domain where high confidence in evidence is expressed as high, moderate confidence, low confidence, and very low.

© 2023 Kastora SL et al. *JAMA Network Open*.

eTable 8. CiNEMa NMA Ratings

Comparison	Number of studies	Within-study bias	Reporting bias	Indirectness	Imprecision	Heterogeneity	Incoherence	Confidence rating	Reason(s) for downgrading
BCCT.core: PROM	3	No concerns	Some concerns	No concerns	No concerns	Major concerns	No concerns	High	["Reporting bias", "Heterogeneity"]
BCCT.core: Panel	4	No concerns	Some concerns	No concerns	Major concerns	No concerns	No concerns	High	["Reporting bias", "Imprecision"]
Panel: PROM	7	No concerns	Some concerns	No concerns	No concerns	Major concerns	No concerns	High	["Reporting bias", "Heterogeneity"]

© 2023 Kastora SL et al. *JAMA Network Open*.

eAppendix. Expert Panel Questionnaire

Different aesthetic assessment modalities following breast cancer therapy

Introduction & Instructions. Dear Colleagues,

Thank you for accepting to be a part of this project. You have been selected on grounds of expertise and engagement in the topic of assessment of aesthetic outcomes (AO) after locoregional breast cancer therapy.

The assessment of AO has been identified as a significant priority in clinical practice and research in recent years. Different modalities have been developed, with Patient-Reported Outcomes (PRO), external validation by a Panel and Software-based assessment being the most popular. However, these modalities are conceptually different. In order to assess the outcomes of the published literature, we conducted a systematic review and network meta-analysis of studies that compared at least two different modalities. You have already received the results in a separate file.

We now ask your contribution to interpret these results. The following questions will be part of the results and will allow us to format the discussion. For questions 2-16, please provide YOUR opinion, regardless of the findings in the meta-analysis. For items with ranking, please "drag" the alternatives from top to bottom (most important to least important). With regards to question 17, we ask for your personal thoughts on the findings of the meta-analysis. Finally, in question 18, we ask for your opinion, based on expertise and the present findings.

Required

1.Please provide your name and affiliation

2.Could a modality replace another in the assessment of AO?

Yes; PRO instead of Panel

Yes; PRO instead of Software

Yes; PRO instead of Panel and Software

Yes; Panel instead of PRO

Yes; Panel instead of Software

Yes; Panel instead of PRO and Software

Yes; Software instead of PRO

Yes; Software instead of Panel

Yes; Software instead of Panel and PRO

No; all are needed

3.Do you feel that different AO modalities “serve the same purpose”?

Yes

No

© 2023 Kastora SL et al. *JAMA Network Open*.

4.If you responded "No" to Question 2, please explain

5.In your opinion, which modalities have a higher degree of overlapping?

PRO and Panel

PRO and software

Panel and software

6. In your opinion, is it possible that the evaluation of AO in PROs is affected by other, “unseen” factors (postoperative pain, chest wall and upper limb morbidity, adverse effects from systemic treatment)?

Yes

No

7.In your opinion, which modality is the most appropriate baseline to guide preoperative decisions regarding type of surgical technique? (rank from most to least important)

PRO

Panel

Software

8.In your opinion, which modality is the most appropriate to evaluate the outcome of primary surgery? (rank from most to least important)

PRO

Panel

Software

9.In your opinion, which modality is the most appropriate to evaluate the outcomes of postoperative radiotherapy? (rank from most to least important)

PRO

Panel

Software

10.In your opinion, which modality is the most appropriate baseline to guide decisions regarding revision surgery? (rank from most to least important)

PRO

Panel

Software

11.In your opinion, which modality is the most appropriate to evaluate outcomes after revision surgery? (rank from most to least important)

PRO

© 2023 Kastora SL et al. *JAMA Network Open*.

Panel

Software

12.In your opinion, which modality is the most appropriate to report AO in clinical routine? (rank from most to least important)

PRO

Panel

Software

13.In your opinion, which modality is the most appropriate to report AO in research? (rank from most to least important)

PRO

Panel

Software

14.In your opinion, which modality is the most appropriate for trainees to understand and assess AO during residency or a fellowship? (rank from most to least important)

PRO

Panel

Software

15. If you feel that there a need for improvement of AO reporting, please provide with the alternatives you feel suit best (more than one alternatives are allowed)

PROs need to develop further and utilise a single type of questionnaire

Panel-based AO evaluation needs standardisation

Software-based standardisation needs to become “smart” (ie, integrate PRO outcomes and/or panel assessments)

16.If you had a SINGLE modality to follow AO, which one would you prefer?

PRO

Panel

Software

17.How would you, as a clinician, interpret the discordance among modalities found in the meta-analysis?

18.Please provide what you would consider as knowledge gaps and research priorities. (Free text answer)

© 2023 Kastora SL et al. *JAMA Network Open*.

eReferences

1. Brunault, P., Suzanne, I., Trzepidur-Edom, M. Depression is associated with some patient-perceived cosmetic changes, but not with radiotherapy-induced late toxicity, in long-term breast cancer survivors. Psycho-Oncology. 2013; 22(3), 590-597.
2. Dahlbäck C, Ringberg A, Manjer J. Aesthetic outcome following breast-conserving surgery assessed by three evaluation modalities in relation to health-related quality of life. Journal of British Surgery. 2019;106(1):90-9.
3. Haloua MH, Krekel NM, Jacobs GJ. Cosmetic outcome assessment following breast-conserving therapy: a comparison between BCCT. core software and panel evaluation. International journal of breast cancer. 2014;1;2014.
4. Hennigs A, Biehl H, Rauch G. Change of patient-reported aesthetic outcome over time and identification of factors characterizing poor aesthetic outcome after breast-conserving therapy: long-term results of a prospective cohort study. Annals of surgical oncology. 2016 May;23(5):1744-51.
5. Kim MK, Kim T, Moon HG.. Effect of cosmetic outcome on quality of life after breast cancer surgery. EJSO. 2015; 1;41(3):426-32.
6. Waljee JF, Hu ES, Ubel PA, Smith DM, Newman LA, Alderman AK. Effect of esthetic outcome after breast-conserving surgery on psychosocial functioning and quality of life. Journal of Clinical Oncology. 2008; 10;26(20):3331-7.
.
7. Sneeuw KC, Aaronson NK, Yarnold JR, Broderick M, Regan J, Ross G, Goddard A. Cosmetic and functional outcomes of breast conserving treatment for early stage breast cancer. 1. Comparison of patients' ratings, observers' ratings and objective assessments. Radiotherapy and oncology. 1992; 1;25(3):153-9.
8. Santos G, Urban C, Edelweiss MI. Long-term comparison of aesthetical outcomes after oncoplastic surgery and lumpectomy in breast cancer patients. Annals of surgical oncology. 2015; 22(8):2500-8.
9. Wu SS, Duraes EF, Scomacao I, Morisada M. Beauty Is in the Eye of the Beholder: Factors Influencing Disparity in Perceptions of Breast Reconstruction Aesthetic Outcomes. Plastic and Reconstructive Surgery. 2022 May 2:10-97
10. Zwakman M, Tan A, Boersma C. Long-term quality of life and aesthetic outcomes after breast conserving surgery in patients with breast cancer. European Journal of Surgical Oncology. 2022 Feb 12.

© 2023 Kastora SL et al. *JAMA Network Open*.
