# Supplementary material for: Outcomes of Different Quality of Life Assessment Modalities After Breast Cancer Therapy: A Network Meta-analysis
Source: JAMA Netw Open. 2023 Jun 6;6(6):e2316878. doi: 10.1001/jamanetworkopen.2023.16878 (PMC10245190; doi:10.1001/jamanetworkopen.2023.16878)
Supplement: Supplement 2. — Data Sharing Statement [file jamanetwopen-e2316878-s002.pdf]

Data Sharing Statement

Kastora. Outcomes of Different Quality of Life Assessment Modalities After Breast Cancer Therapy. *JAMA Netw Open*. Published June 06, 2023.
doi:10.1001/jamanetworkopen.2023.16878

Data
Data available: Yes
Data types: Other (please specify)
Additional Information: Included studies aggregate data
How to access data: As supplementary material in the online version of the publication
When available: With publication

Supporting Documents
Document types: None

Additional Information
Who can access the data: anyone requesting the data
Types of analyses: any further analyses
Mechanisms of data availability: As supplementary material in the online version of the publication
